# The penetrance of rare variants in cardiomyopathy-associated genes: A cross-sectional approach to estimating penetrance for secondary findings

**DOI:** 10.1016/j.ajhg.2023.08.003

**Published:** 2023-08-30

**Authors:** Kathryn A. McGurk, Xiaolei Zhang, Pantazis Theotokis, Kate Thomson, Andrew Harper, Rachel J. Buchan, Erica Mazaika, Elizabeth Ormondroyd, William T. Wright, Daniela Macaya, Chee Jian Pua, Birgit Funke, Daniel G. MacArthur, Sanjay K. Prasad, Stuart A. Cook, Mona Allouba, Yasmine Aguib, Magdi H. Yacoub, Declan P. O'Regan, Paul J.R. Barton, Hugh Watkins, Leonardo Bottolo, James S. Ware

**Affiliations:** 1 National Heart and Lung Institute, Imperial College London, London, UK; 2 MRC London Institute of Medical Sciences, Imperial College London, London, UK; 3 Royal Brompton & Harefield Hospitals, Guy’s and St. Thomas’ NHS Foundation Trust, London, UK; 4 Division of Cardiovascular Medicine, Radcliffe Department of Medicine and the Wellcome Trust Centre for Human Genetics, University of Oxford, Oxford, UK; 5 Northern Ireland Regional Genetics Centre, Belfast Health and Social Care Trust, Belfast City Hospital, Belfast, Northern Ireland, UK; 6 GeneDx LLC, Gaithersburg, MD, USA; 7 National Heart Research Institute Singapore and Duke-National University of Singapore, Singapore, Singapore; 8 Laboratory for Molecular Medicine, Partners Healthcare Center for Personalized Genetic Medicine, Boston, MA, USA; 9 Centre for Population Genomics, Garvan Institute of Medical Research and UNSW, Sydney, NSW, Australia; 10 Centre for Population Genomics, Murdoch Children’s Research Institute, Melbourne, VIC, Australia; 11 Aswan Heart Centre, Aswan, Egypt; 12 Department of Medical Genetics, University of Cambridge, Cambridge, UK; 13 The Alan Turing Institute, London, UK; 14 MRC Biostatistics Unit, University of Cambridge, Cambridge, UK

**Keywords:** penetrance, cardiomyopathy, prevalence, secondary findings

## Abstract

Understanding the penetrance of pathogenic variants identified as secondary findings (SFs) is of paramount importance with the growing availability of genetic testing. We estimated penetrance through large-scale analyses of individuals referred for diagnostic sequencing for hypertrophic cardiomyopathy (HCM; 10,400 affected individuals, 1,332 variants) and dilated cardiomyopathy (DCM; 2,564 affected individuals, 663 variants), using a cross-sectional approach comparing allele frequencies against reference populations (293,226 participants from UK Biobank and gnomAD). We generated updated prevalence estimates for HCM (1:543) and DCM (1:220). In aggregate, the penetrance by late adulthood of rare, pathogenic variants (23% for HCM, 35% for DCM) and likely pathogenic variants (7% for HCM, 10% for DCM) was substantial for dominant cardiomyopathy (CM). Penetrance was significantly higher for variant subgroups annotated as loss of function or ultra-rare and for males compared to females for variants in HCM-associated genes. We estimated variant-specific penetrance for 316 recurrent variants most likely to be identified as SFs (found in 51% of HCM- and 17% of DCM-affected individuals). 49 variants were observed at least ten times (14% of affected individuals) in HCM-associated genes. Median penetrance was 14.6% (±14.4% SD). We explore estimates of penetrance by age, sex, and ancestry and simulate the impact of including future cohorts. This dataset reports penetrance of individual variants at scale and will inform the management of individuals undergoing genetic screening for SFs. While most variants had low penetrance and the costs and harms of screening are unclear, some individuals with highly penetrant variants may benefit from SFs.

## Graphical abstract

**Figure:**
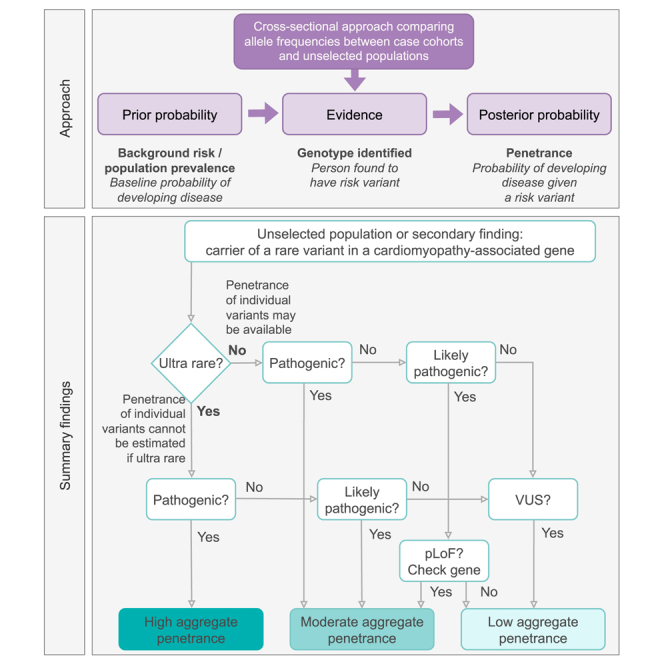

## Teaser

The importance of estimating the penetrance of individual variants, i.e., the probability of developing disease given a DNA variant, to guide intervention is ever increasing. We undertake a cross-sectional approach, meta-analyzing unique, large cardiomyopathy referral cohorts and leveraging publicly available population-based cohorts to estimate variant-specific penetrance for rare CM-associated variants.

## Introduction

Cardiomyopathies (CMs) are diseases of the heart muscle, characterized by abnormal cardiac structure and function that is not due to coronary disease, hypertension, valve disease, or congenital heart disease. Many affected individuals have a monogenic etiology with autosomal dominant inheritance. Penetrance is incomplete and age related, and expressivity is highly variable. These features present huge challenges for disease management. In particular, the penetrance of variants in CM-associated genes is incompletely characterized and poorly understood, especially when identified in an asymptomatic individual without family history of CM. With the growing availability of exome and genome sequencing in wider clinical settings and consumer-initiated elective genomic testing,^1^ the importance of estimating the penetrance of individual variants identified as secondary findings (SFs) to guide intervention is ever increasing.

SFs are genetic variants that are actively sought out (as opposed to incidental findings) but that are unrelated to the clinical indication for genetic testing and can therefore be considered as opportunistic genetic screening. Genes associated with inherited CMs make up one-fifth of the 78 genes recommended by the American College of Medical Genetics and Genomics (ACMG SF v.3.1) for reporting SFs during clinical sequencing.^2^ It is recommended to return variants that would be classified as pathogenic or likely pathogenic in an affected individual with >90% confidence that the variant is causing the observed disease. This is independent of the probability that an individual carrying the variant will develop disease (penetrance). The ACMG SF guidelines have not yet been adopted globally; the European Society of Human Genetics recommends a cautious approach but is responsive to accumulating evidence.^3^^,^^4^

We are concerned that the costs, harms, and benefits have not been fully characterized. We have previously discussed issues with the recommendations based on the lack of estimates of the harms and cost of this approach for variants in specific genes.^5^ These estimates are required to conform to the ninth rule of Wilson and Jungner’s principles of screening.^6^ The burden of the implementation of reporting SFs in specific healthcare systems remains unassessed. There is little evidence for clinical utility and limited justification for use of resources.^4^ Research is beginning to become available on implementation frameworks^7^ and the perspectives of and impact on individuals with disease.^8^^,^^9^^,^^10^^,^^11^^,^^12^

Subclinical phenotypic expressivity of rare variants in CM-associated genes has been demonstrated in the UK Biobank (UKBB) population cohort.^13^^,^^14^^,^^15^ Causes of variability in penetrance may include (1) genetic and allelic heterogeneity, as different alleles have different consequences on protein function; (2) environmental modifiers altering genetic influence (e.g., age, sex, hypertension, lifestyle); and (3) additional genetic modifiers with additive or epistatic interactions with the variant of interest (other variants or combinations of genetic factors, e.g., polygenic risk, variants in *cis* that drive allelic imbalance, imprinting, epigenetic regulation, compensation, threshold model, and transcript isoform expression).^16^^,^^17^^,^^18^^,^^19^^,^^20^^,^^21^^,^^22^

Variant-specific estimates of penetrance are required to appropriately inform clinical practice and to fully utilize genetics as a tool to individualize the risk of developing disease in asymptomatic heterozygotes.^5^^,^^23^ It is challenging to estimate the penetrance of individual rare variants through other study methods, as longitudinal population studies require very large sample sizes and long-term follow-up is required if penetrance is age related. Where data are available for rare variants in CM-associated genes, reported penetrance is mostly estimated from family-based studies. These may be affected by ascertainment biases and secondary genetic and environmental factors^24^ and thus less applicable to SFs. Penetrance has been estimated in aggregate by gene and by disease.^13^^,^^25^^,^^26^ Variant-specific penetrance in the general adult population for rare variants in CM-associated genes is unknown.

Here, we apply a cross-sectional approach by using a method^26^ that compares the allele frequency of individual rare variants in large cohorts of phenotypic affected individuals with the background frequency of the same variants in the population (phenotype agnostic) to estimate penetrance. As well as providing aggregate penetrance estimates for groups of rare variants (e.g., those curated as pathogenic), this approach can estimate the penetrance of *individual* rare alleles. Importantly, these estimates represent variants in the general population rather than in families ascertained for disease.

## Subjects and methods

### Case cohort

Sequencing data for 10,400 individuals referred for hypertrophy cardiomyopathy (HCM) gene panel sequencing and 2,564 individuals referred for dilated cardiomyopathy (DCM) gene panel sequencing was collected from seven international testing centers: three UK-based centers—the NIHR Royal Brompton Biobank, Oxford Molecular Genetics Laboratory, and Belfast Regional Genetics Laboratory; two US-based centers—the Partners Laboratory of Molecular Medicine and GeneDx; the National Heart Centre, Singapore; and Aswan Heart Centre, Egypt. Although the diagnosis cannot directly be reconfirmed, given genetic testing guidelines (e.g., Wilde et al.,^27^ Ackerman et al.^28^), a clinical diagnosis of CM is implicit. For information on DNA sequencing and data obtained for analyses, see the supplemental information.

For each variant observed in one or more individuals referred for CM sequencing, we calculated the allele count (AC) and allele number (AN) and further stratified by reported age, sex, and ancestry where the data allowed. All research participants provided written informed consent, and the studies were reviewed and approved by the relevant research ethics committee (Aswan Heart Centre: FWA00019142, research ethics committee code 20130405MYFAHC_CMR_20130330; NIHR Royal Brompton Biobank: South Central – Hampshire B Research Ethics Committee, 09/H0504/104+5, 19/SC/0257; National Heart Centre Singapore: Singhealth Centralised Institutional Review Board 2020/2353 and Singhealth Biobank Research Scientific Advisory Executive Committee SBRSA 2019/001v1; UK Biobank: National Research Ethics Service 11/NW/0382, 21/NW/0157, under terms of access approval number 47602).

In addition, diagnostic laboratories (Oxford Molecular Genetics Laboratory, Belfast Regional Genetics Laboratory, the Partners Laboratory of Molecular Medicine, and GeneDx) provided aggregated (and therefore fully anonymous) cohort-level summaries of variant data collected for clinical purposes during routine healthcare. Secondary use of this data did not require research consent from individuals, and approval for public release of the data followed local governance procedures. Data are publicly available through DECIPHER (https://www.deciphergenomics.org/). Analyses of these data do not require research ethics committee approval.

### Population cohort

167,478 participants of the UK Biobank (UKBB) with whole-exome-sequencing data available for analyses and 125,748 exome sequenced participants of the Genome Aggregation Database (gnomAD; version v.2.1.1) were included in this study.

Briefly, the UKBB recruited participants aged 40–69 years old from across the UK between 2006 and 2010,^29^ of which the 200,571 exome tranche of individuals that had not withdrawn were included in this study.^30^ The maximal subset of unrelated participants was used, identified by those included in the UKBB principal-component analysis (PCA) (S3.3.2,^29^ n = 167,478). Age at recruitment, genetic sex, and genetic (for European [EUR] and British ancestry) or reported ancestry information (for other global ancestries: AFR, African, Caribbean [n = 2,903]; SAS, Indian, Pakistani, Bangladeshi [n = 3,136]; EAS, Chinese [n = 605]) were incorporated.

gnomAD contains sequencing information for unrelated individuals sequenced as part of various disease-specific and population genetic studies.^31^ The version 2 short variant dataset spans 125,748 exomes. We used Ensembl Variant Effect Predictor^32^ (VEP, version 105) to incorporate the variant-specific summary counts. Variants flagged by gnomAD as AC0 were excluded from gnomAD counts. For more information on the incorporation of these datasets, please see the supplemental information.

### Variant annotation

We used VEP (105) to annotate the case and population datasets, with additional plugins: gnomAD^31^ (version r2.1), LOFTEE,^31^ SpliceAI^33^ (1.3.1), REVEL^34^ (1.3), and ClinVar^35^ (20220115). The data were organized with PLINK^36^ (1.9) and the VEP output was analyzed with R (4.1.2).

Protein-altering variants, defined with respect to MANE transcripts, that were annotated as high or moderate impact by Sequence Ontology and Ensembl were included in the analysis. We restricted the analysis to genes with strong or definitive evidence of causing CM following ClinGen guidance^37^^,^^38^ and expert curation^39^ to include eight sarcomeric HCM-associated genes (HCM [MIM: 192600]: *MYH7* [MIM: 160760], *MYBPC3* [MIM: 600958], *MYL2* [MIM: 160781], *MYL3* [MIM: 160790], *ACTC1* [MIM: 102540], *TNNI3* [MIM: 191044], *TNNT2* [MIM: 191045], *TPM1* [MIM: 191010]) and 11 DCM-associated genes (DCM [e.g., MIM: 613426 and 604145]: *BAG3* [MIM: 603883], *DES* [MIM: 125660], *DSP* [MIM: 125647], *LMNA* [MIM: 150330], *MYH7* [MIM: 160760], *PLN* [MIM: 172405], *RBM20* [MIM: 613171], *SCN5A* [MIM: 600163], *TNNC1* [MIM: 191040], *TNNT2* [MIM: 191045], *TTN*_*PSI > 90%*_ [MIM: 188840]), with the exception of *FLNC* [MIM: 102565], which was not included on the panel sequencing of the DCM case cohort (Table S4). Variants with consequences consistent with the known disease-causing mechanism were retained.

Further manual annotation was undertaken following ACMG guidelines with ClinVar^35^ and Cardioclassifier,^40^ as previously published.^13^ For analyses of variants in aggregate, the UKBB data were filtered following the same thresholds and used to estimate aggregate penetrance.

### Statistical analysis

#### Estimation of penetrance and 95% confidence interval

Penetrance, the probability of a disease given a risk allele, is expressed as a probability function on a scale of 0–1 or as a percentage. Penetrance was estimated from case-population data in a Binomial framework following Bayes’ theorem^26^

$$P\left(D|A\right)=P\left(D\right)\frac{P\left(A|D\right)}{P\left(A\right)}$$

$$penetrance=population\;prevalence\frac{case\;allele\;frequency}{population\;allele\;frequency}$$

where, D, disease; A, allele; P, probability; P(D|A) = penetrance (probability of disease given a risk allele), P(D) = prevalence, the population baseline risk of disease (probability of disease); P(A|D) = allele frequency in the case cohort (probability of the allele given disease); and P(A) = allele frequency in the population cohort (probability of the allele).

We define penetrance in this setting as the probability of dominant CM by late adulthood (UKBB had a mean age of 56 years old at recruitment). We assume the independence of the random variables in the penetrance equation above to derive the 95% confidence interval for penetrance as the product and ratio of binomial proportions. We used the specialized version of the central limit theorem, the delta method, on the log-transformed random variable log(D|A)=log(D)+log(A|D)−log⁡(A) with an improved mean approximation and adjustment for degeneracy (as allele frequency tends to 0 for rare variants). Please see additional methods and alternative approaches considered (supplemental methods, Table S3; Figures S4 and S5).

For estimates of penetrance by sex, we adjusted all terms of the penetrance equation by values for sex-specific parameters. For estimates of penetrance by ancestry, we kept P(D) as estimated for CM (there are few estimates of the prevalence of CM in specific ancestries) and proportioned P(A|D) and P(A) by reported ancestry. For estimates of penetrance by age, we normalized P(D) by the number diagnosed in the case cohort by a particular age in a cumulative fashion, with P(A|D) by a particular age and P(A) fixed as total population allele frequency (supplemental methods).

### Estimated cardiomyopathy prevalence

To incorporate P(D) in our penetrance analysis, we estimated the uncertainty surrounding the reported prevalence of CM (Tables S1 and S2; Figures S1–S3). For HCM, we meta-analyzed four imaging-based prevalence estimates^13^^,^^41^^,^^42^^,^^43^ excluding studies with potential selection biases. From the meta-analysis estimate ($p_{D}\equiv P\left(D\right)$) and its confidence interval, we derived values of allele count, $x_{D}$, and allele number, $n_{D}$ (where $p=\frac{x}{n}$). A literature review was also completed for DCM, but there were not enough imaging-based prevalence estimates in literature, so we used 39,003 participants of the UKBB imaging cohort to estimate phenotypic DCM^44^^,^^45^^,^^46^ (supplemental methods). Using the same methods and included studies, we derived estimates for male- and female-specific HCM and DCM prevalence.

## Results

### Case cohort summary information

Sequencing data for 10,400 individuals referred for HCM genetic panel sequencing and 2,564 individuals referred for DCM genetic panel sequencing were included in the analysis. Aggregate frequency of rare protein-altering variants in well-established disease-associated genes was 41% for HCM and 32% for DCM in the respective case cohorts (Tables S6 and S7). Of the cohorts with age, sex, and ancestry information available (20% of HCM-affected individuals, 42% of DCM-affected individuals), 35% and 32% were female, 93% and 91% were of EUR ancestry, and mean age was 48 and 49 years old, for HCM and DCM, respectively (Table S5).

### Estimates of the prevalence of CMs

To estimate the prevalence of CMs, we undertook a literature review and meta-analysis (Tables S1 and S2; Figures S1–S3). Prevalence is underestimated when derived from national cohorts using coding systems such as ICD codes because of incomplete ascertainment through diagnostic and procedure coding.^47^ We would therefore expect the most accurate estimates of the prevalence of CM to come from imaging studies in populations, where echocardiogram or cardiac magnetic resonance imaging was used to identify CM within a population sample that is representative. The estimates are not generalizable if the prevalence is estimated for selected subgroups of individuals, such as young, elderly, or athletic cohorts. We therefore meta-analyzed four imaging-based prevalence estimates, which resulted in an HCM population prevalence estimate of 1 in 543 individuals ($p_{D}=$ 0.18% [95% ${CI}_{D}=$ 0.15%–0.23%]).^13^^,^^41^^,^^42^^,^^43^ The well reported estimate of 1 in 500 individuals for HCM prevalence (0.20%) is within this confidence interval.

A literature review revealed insufficient imaging-based estimates to undertake a direct meta-analysis of the prevalence of DCM. Instead, we used 39,003 participants of the UKBB imaging cohort to estimate phenotypic DCM.^44^^,^^45^^,^^46^ This derived a DCM population prevalence of 1 in 220 individuals ($p_{D}=$ 0.45% [95% ${CI}_{D}=$ 0.39%–0.53%]), which includes the well reported estimate of 1 in 250 (0.40%)^48^ within the confidence interval.

We also estimated sex-specific CM prevalence. This resulted in an HCM population prevalence of ∼1 in 1,300 females ($p_{D}=$ 0.08% [95% ${CI}_{D}=$ 0.04%–0.12%]) and ∼1 in 360 males ($p_{D}=$ 0.28% [95% ${CI}_{D}=$ 0.22%–0.35%]) and a DCM population prevalence of ∼1 in 340 females ($p_{D}=$ 0.30% [95% ${CI}_{D}=$ 0.23%–0.38%]) and ∼1 in 160 males ($p_{D}=$ 0.63% [95% ${CI}_{D}=$ 0.52%–0.75%]).

### Estimated penetrance of rare variants in aggregate

In individuals with cardiomyopathy referred for diagnostic sequencing, we identified 1,332 rare (inclusive population allele frequency of <0.1%) variants in HCM-associated genes (4,305 observations, case frequency 41%) and 663 rare variants in DCM-associated genes (831 observations, case frequency 32%) (Tables S6–S9). The UKBB dataset was filtered following the same pipeline. We used 1,719 rare variants in HCM-associated genes (9,152 observations, 5.5% population frequency) and 4,568 rare variants in DCM-associated genes (22,177 observations; 13.2% population frequency) to estimate penetrance of rare variant subgroups in aggregate.

**Figure 1:**
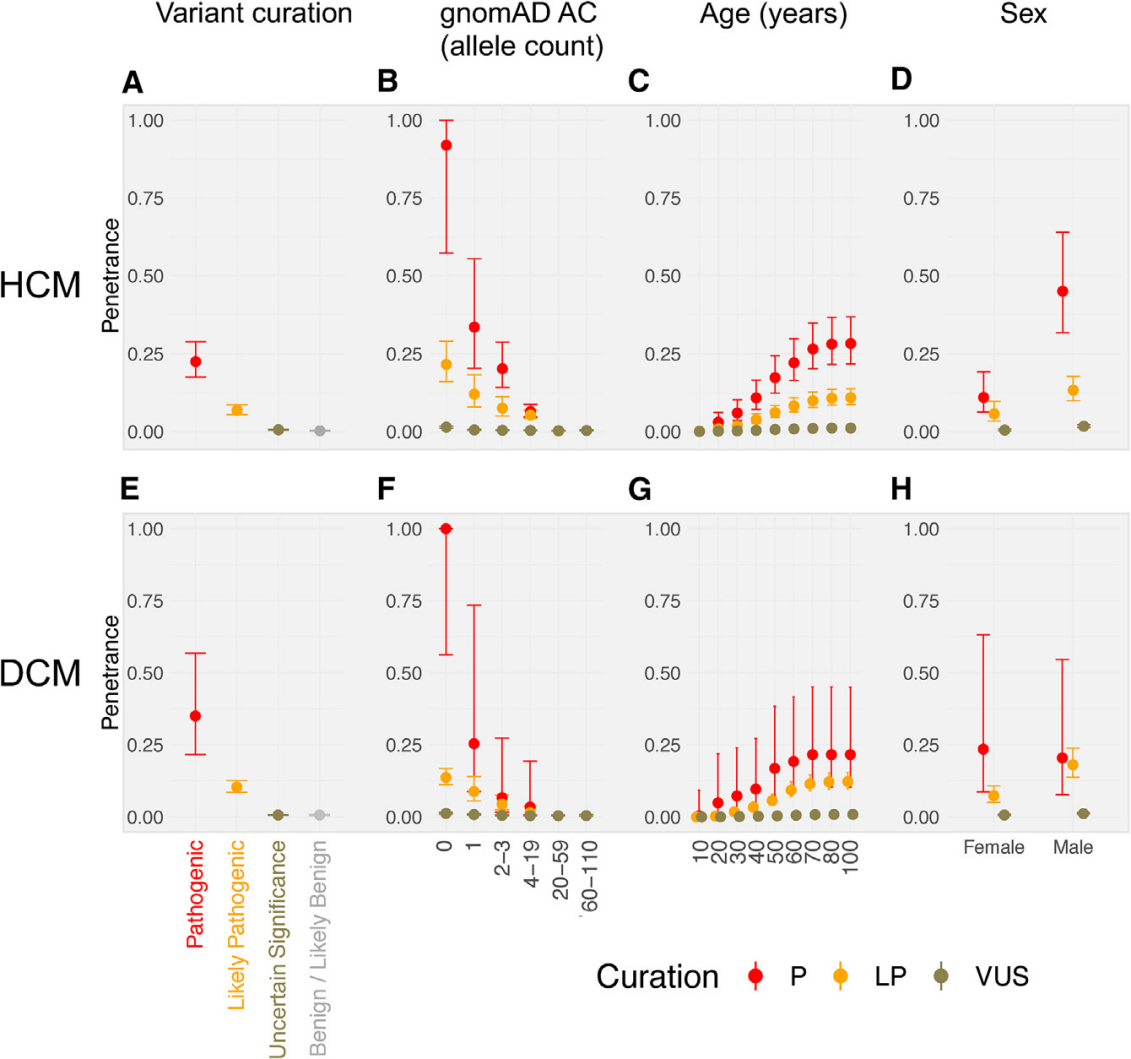
Penetrance of rare variants in aggregate by variant curation, rarity, age, and sex (A–H) In aggregate, variants curated as pathogenic and variants that are particularly rare (gnomAD allele count [AC] = 0) were most penetrant. The plot depicts aggregate estimated penetrance and 95% confidence intervals for rare variants in HCM- (A, B, C, D) and DCM-associated (E, F, G, H) genes. Variant curation was assessed following ACMG guidelines through ClinVar and CardioClassifier software with additional manual curation of variants with conflicting evidence (A and E [for HCM affected individuals: 173 P variants, 316 LP, 824 VUSs, 19 LB; for the UK Biobank: 30 P, 97 LP, 1,536 VUSs, 54 LB, 2 B; for DCM affected individuals: 21 P, 245 LP, 356 VUSs, 37 LB, 4 B; for the UK Biobank: 15 P, 505 LP, 3,933 VUSs, 108 LB, 7 B]). The variants were assessed for rarity by gnomAD AC bins, where 0 is not identified in the gnomAD dataset (B and F). Age was assessed in decades based on the cumulative proportion of affected individuals analyzed by each age timepoint (C and G). Sex was estimated with all parameters stratified by reported sex (D and H).

Variants with a pathogenic classification in ClinVar were the most penetrant subgroup by ACMG classification^45^ (HCM 22.5% [17.5%–28.8%], DCM 35.0% [21.6%–56.8%]; Figure 1, Table S15). An estimate of the aggregate penetrance of both pathogenic and likely pathogenic variants in HCM was 10.7% (8.7%–13.3%) with this approach, concordant with a recent estimate derived via direct assessment of cardiac imaging in UKBB (10.8%; individuals with variants and left ventricular hypertrophy (LVH) ≥ 13mm without hypertension or valve disease; binomial 95% confidence interval of 3.0%–25.4%; n = 4/37).^10^ This concordance was also observed for other variants in the same paper (e.g., VUSs), for which we estimated penetrance as 0.55% (0.45%–0.68%) compared to 0.57% (0.07%–2.03%, n = 2/353).^10^

**Figure 2:**
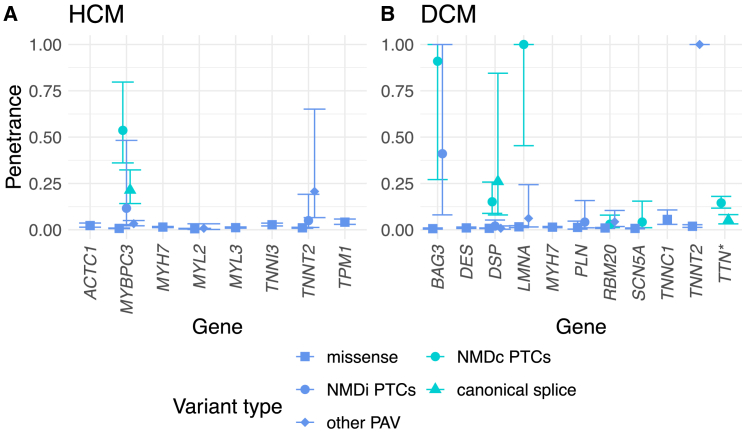
The aggregate estimates of penetrance of loss-of-function variants are high for specific genes The plot depicts estimated penetrance and 95% confidence interval of HCM-associated (A) and DCM-associated (B) rare variants. Predicted loss-of-function (pLoF) and non-pLoF variant groups are plotted in green and blue, respectively. ^∗^, TTNtvs that are PSI > 90%. Pathogenic *TNNT2* inframe deletions caused an increased penetrance signal for inframe deletions for both HCM and DCM (see Figure S12). PTC, premature termination codon; PAV, protein-altering variant; NMDc/NMDi, nonsense mediated decay competent/incompetent.

The aggregate penetrance of pathogenic and likely pathogenic variants in DCM was 11.3% (9.3%–13.6%). Population penetrance of rare variants in DCM-associated genes in UKBB has been previously estimated as ≤30%^49^ for a clinical or subclinical diagnosis in an analysis of 44 DCM-associated genes and in the range of 5%–6% for truncating variants in *TTN* (TTNtvs, 1.9%–12.8%; 877 individuals with variants)^5^ depending on the definition used. We report a concordant penetrance estimate from our analysis of strong and definitive evidence DCM-associated genes only and 9.8% (8.0%–12.1%) for all TTNtvs (Figures 2 and S12).

Variants predicted to result in premature termination codons (PTCs; nonsense-mediated decay competent or incompetent^50^) in *MYBPC3*, *BAG3*, *DSP*, and *LMNA* were the most penetrant. Inframe deletions in *TNNT2* were highly penetrant for both HCM and DCM. TTNtvs and missense variants predicted to be damaging in *TPM1* and *TNNC1* had moderate penetrance (Figures 2 and S12; Tables S13, S14, S18, and S19).

Stratification by variant rarity showed that variants absent from gnomAD were the most penetrant subgroup (HCM pathogenic 91.9% [57.3%–100.0%], HCM likely pathogenic 22.1% [16.4%–29.8%], DCM pathogenic 100.0% [56.3%–100.0%], DCM likely pathogenic 13.7% [11.2%–16.8%]; Figure 1, Table S16). Stratification of penetrance by sex identified increased penetrance for males compared to females for rare variants in HCM-associated genes (Figures 1 and S13; Table S20). We estimated penetrance as <20% up to 50 years of age by modeling the penetrance of CM as an age-related cumulative frequency by using the proportion of affected individuals referred at each age decile (Figure 1; Table S17).

While there are limitations to the cohort size when split by reported ancestry and we are unable to rule out local ancestry mismatches between case and population datasets, there was no significant difference in the penetrance of TTNtvs between African (5.7% [2.9%–10.9%]), European (6.9% [5.5–8.5%]), East Asian (6.1% [3.0%–12.4%]), and South Asian (5.7% [2.1%–15.8%]) ancestries, as previously suggested.^51^

### Estimated penetrance of individual rare variants

**Figure 3:**
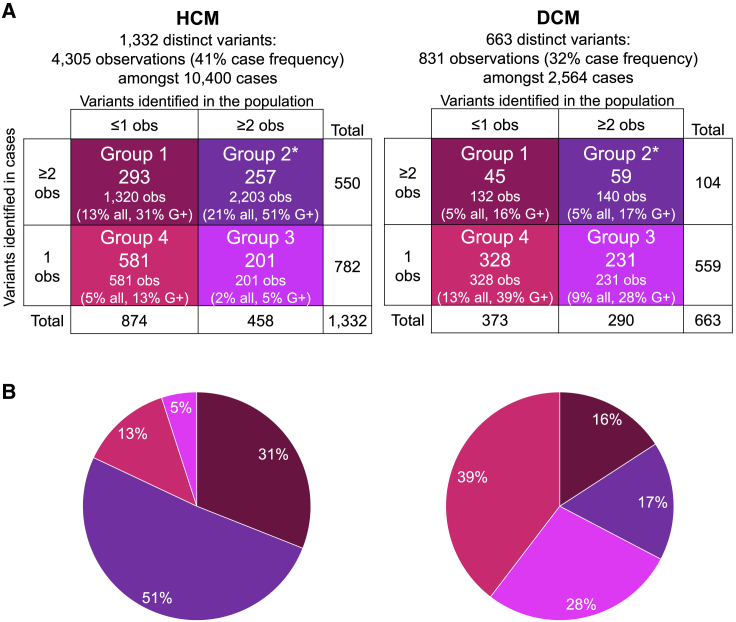
Penetrance of individual variants could be estimated for 316 recurrently observed rare variants from group 2 (A) The figure shows variant counts and subgroups for rare variants in HCM-associated (left) and DCM-associated (right) genes. (B) The pie charts plot the proportion of all variant observations in each subgroup (also denoted as “G+”). The observations approximate to the number of individuals with variants, although a small number of individuals may carry more than one variant. All, denotes frequency of the variant in affected individuals; obs, denotes observations of allele count. Group 1: variants observed recurrently in affected individuals and absent or singleton in the population; penetrance estimates are unreliable as the population frequency is uncertain. This group is expected to include most definitively pathogenic, high-penetrance variants. Group 2^∗^: variants observed recurrently in affected individuals and the wider population; these are the variants most likely to be observed as secondary findings. ^∗^Penetrance can be estimated. Group 3: variants observed once in affected individuals and recurrently in the population; penetrance estimates are unreliable, as the case frequency is uncertain. Variants in this group are likely either not pathogenic or have low penetrance. Group 4: variants are singleton in affected individuals and absent or singleton in the population; current data is too sparse to estimate penetrance.

Of the variants identified and used to estimate penetrance in aggregate, we report four subgroups of variants in our case series (Figure 3):

Group 1 consisted of 338 variants that were found in more than one affected individual (case allele count [AC] ≥ 2) and were ultra-rare in population reference sets (population AC [pop AC] ≤ 1). Penetrance cannot be estimated with precision for individual variants in this group, since the population allele frequency (AF) cannot be estimated with precision. When considered in aggregate, this group has high penetrance (Figures S14). For HCM, 293 variants in group 1 were identified 1,320 times (13% case frequency, 31% observations). 29% were curated as pathogenic (P, n = 84, 41% of HCM group 1 observations), 34% were likely pathogenic (LP, n = 100, 36% observations), and 37% were curated as uncertain significance (VUSs, n = 109, 23% observations). For DCM, 45 variants in group 1 were identified 132 times (5% case frequency, 16% observations). 18% of these were P (n = 8, 20% DCM group 1 observations), 49% LP (n = 22, 55% observations), and 33% VUSs (n = 15, 25% observations).

**Figure 4:**
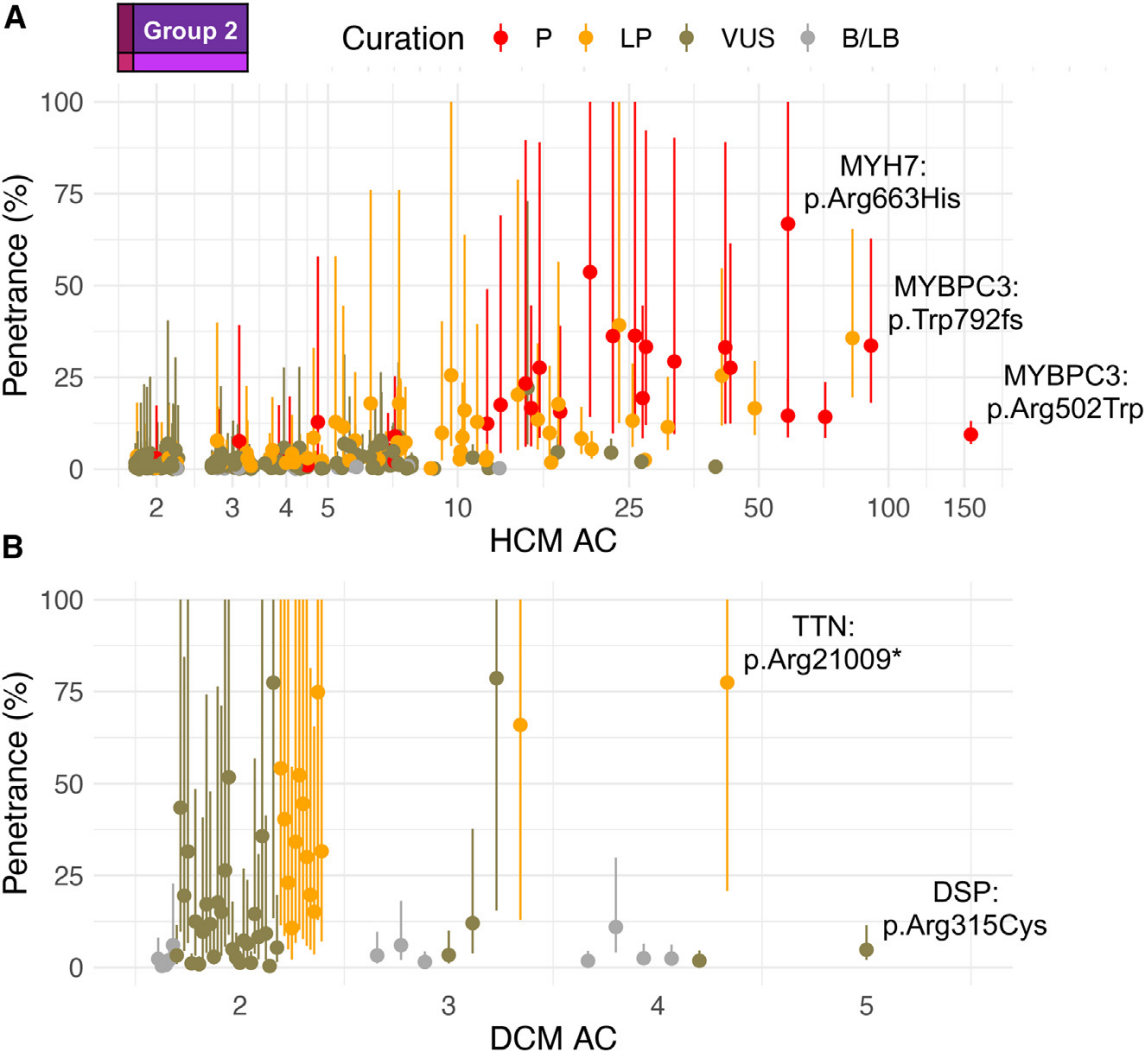
Variant-specific estimates of penetrance for the 316 recurrently observed rare variants in CM-associated genes from group 2 An interactive widget is available for browsing the individual variants in this figure (see Figure S15). The variants depicted (HCM n = 257, A; DCM n = 59, B) were identified multiple times in affected individuals and population reference datasets and penetrance could therefore be estimated. Presented is the estimated penetrance and 95% confidence interval. The x axis denotes the number of times the variant was observed in each case cohort. AC, allele count; B/LB, benign/likely benign; VUS, variant of uncertain significance; LP, likely pathogenic; P, pathogenic.

Group 2 included 316 variants found multiple times in both affected individuals and population reference datasets (case AC ≥ 2, pop AC ≥ 2). This group is expected to include variants with intermediate penetrance, including founder effect variants. For this group, we can estimate AF in both populations and therefore can estimate penetrance (Figure 4, Interactive Figure S15; Tables S10 and S11). These account for more than half of all variants identified in HCM-associated genes and include those most likely to be identified as SFs, as they are identified multiple times in the population. For HCM, 257 variants were identified a total of 2,203 times (21% case frequency, 51% observations). 11% were P (n = 29, 37% HCM group 2 observations), 25% LP (n = 64, 31% observations), 59% VUSs (n = 151, 29% observations), and 5% likely benign (LB, n = 13, 3% observations). 49 of these variants were recurrent at least ten times and described a large portion of observations (case AC ≥ 10; found 1,424 times, 33.0% of case cohort observations, case frequency of 13.7%). The median penetrance of these was 14.6% (±14.4% SD). For DCM, 59 variants were identified 140 times (5% case frequency, 17% observations). None were curated as P, 24% were LP (n = 14, 22% DCM group 2 observations), 56% VUSs (n = 33, 53% observations), 17% LB (n = 10, 21% observations), and 3% B (n = 2, 4% observations). With the current DCM case cohort size, no variant was identified ten or more times.

The final two groups consisted of 1,350 variants with only a single observation in our case series. This does not provide a reliable estimate of case frequency, so penetrance estimates would lack precision. Group 3 variants were those identified multiple times in the population (pop AC ≥ 2) and consisted mostly of VUSs: for HCM, 201 variants were identified (2% case frequency, 5% of case observations). This included 0.5% P (n = 1; *MYBPC3* c.3297dup [p.Tyr1100Valfs^∗^49] [GenBank: NM_000256.3]), 5% LP (n = 10), 92% VUSs (n = 184), and 3% LB (n = 6). For DCM, 231 variants were identified (9% case frequency, 28% observations). 1% were P (n = 3), 7% LP (n = 17), 79% VUSs (n = 182), 12% LB (n = 27), and 1% B (n = 2).

Group 4 variants are those observed once in affected individuals and rarely in the population reference dataset (pop AC ≤ 1). A substantial portion of these were P/LP: for HCM, 583 variants were identified (5% case frequency, 13% observations). 10% were P (n = 59), 24% LP (n = 142), and 66% VUSs (n = 380). For DCM, 328 variants were identified (13% case frequency, 39% observations). 3% were P (n = 10), 59% LP (n = 192), and 38% VUSs (n = 126).

### The impact of age, sex, and ancestry on variant-specific penetrance estimates

**Figure 5:**
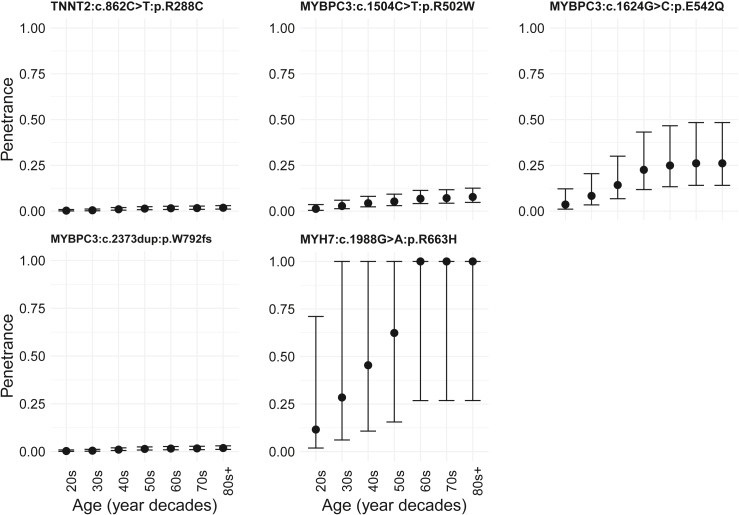
Variant-specific estimates of penetrance by age can now be derived The plot depicts the age-related cumulative penetrance of five HCM-associated rare variants across age deciles from variant group 2. Presented is the estimated penetrance and 95% confidence interval. The x axis starts in the decade of the 20s as the analysis of these variants was underpowered for teens and younger. “20s” here means “by 30 years old.”

For group 2, where age-related penetrance could be derived, we estimated the penetrance of specific variants by decade of age (e.g., Figure 5). For some variants (e.g., *MYBPC3* c.1624G>C [p.Glu542Gln] [GenBank: NM_000256.3]), the age-related penetrance curve shows infrequent onset before middle age. These curves may inform surveillance strategies in individuals with variants unaffected at first assessment.

We identified rare variants in HCM-associated genes where estimated penetrance for males was significantly increased compared to females (Figure S13). Identification of such variants allows for future investigations of modifiers protecting females with variants from disease.

For estimates of penetrance by ancestry, variants that were nominally more common in AFR, EAS, or SAS ancestries compared to EUR ancestry were identified (Table S12). We interpret these as more consistent with an inaccurate penetrance estimation arising from ancestries where the variant is sparsely observed rather than true differences in penetrance on different ancestral background. For example, *MYBPC3* c.1544A>G (p.Asn515Ser) (GenBank: NM_000256.3) was identified 5/492 times in AFR affected individuals (AF = 0.005) and 33/10,655 times in AFR population participants (AF = 0.0016; penetrance of 0.6% [0.2%–1.5%]) compared to 1/9,692 times in EUR affected individuals (AF = 0.00005) and not observed in 211,532 EUR population participants. Even when ancestry is nominally matched, broad continental groupings hide great diversity and results may be misleading due to stratification between case datasets (mostly North AFR from Egypt) and population reference datasets (e.g., UKBB participants from the Caribbean) (Box 1).

Box 1 **Case study: The MYBPC3 c.1504C>T (p.Arg502Trp) Northwestern European variant**

The variant *MYBPC3* c.1504C>T (p.Arg502Trp) (GenBank: NM_000256.3) was found in our cohort 159 times in individuals referred for HCM genetic panel sequencing (3.7% of total observations; 1.5% total case frequency). To date, the variant has been classified on ClinVar 15 times as pathogenic (ClinVar ID 42540). Penetrance has been previously estimated as ∼50% (increased relative risk of 340) by 45 years old in a clinical setting, and major adverse clinical events in heterozygotes are significantly more likely when another sarcomeric variant is present.^52^

In our case cohort, heterozygotes of this variant were reported as broadly European ancestry (Oxford, n = 59; London, n = 11; Belfast, n = 30; LMM, n = 45; GDX, n = 14). In gnomAD, the variant was identified ten times, of which seven heterozygotes were non-Finnish Northwestern Europeans (NWE; plus one African; one South Asian, and one other), and in the UK Biobank, the variant was found 77 times, of which 68 heterozygotes were NWE (plus eight other Europeans and one other). The population frequency of the variant in Ensembl population genetics showed that the variant (rs375882485) is only found multiple times in NWE ancestry sub-cohorts. Thus, the variant is most common in NWE populations: the UK, Ireland, Belgium, the Netherlands, Luxembourg, Northern France, Germany, Denmark, Norway, Sweden, and Iceland.

We use this relatively common variant to highlight the effect of ancestry on estimated variant penetrance (see related figure in this text box):

we estimated the penetrance as 6.4% (4.6%–9.0%) with the UK Biobank cohort (93% European) and this is inflated to 35.1% (18.2%–67.5%) when we estimated the penetrance with the gnomAD dataset (45% European) as a result of the difference in the proportion of individuals with NWE ancestry. In individuals of NWE ancestry only, the penetrance of this variant is 6.4% (4.6%–9.0%). Penetrance estimated from the NWE subset of gnomAD or UKBB do not differ significantly.

As access to larger genomic datasets becomes available, including more diverse ancestries, we can increase the precision of these variant-specific penetrance estimates by gaining further confidence in maximum population allele frequencies.^53^

**Figure:**
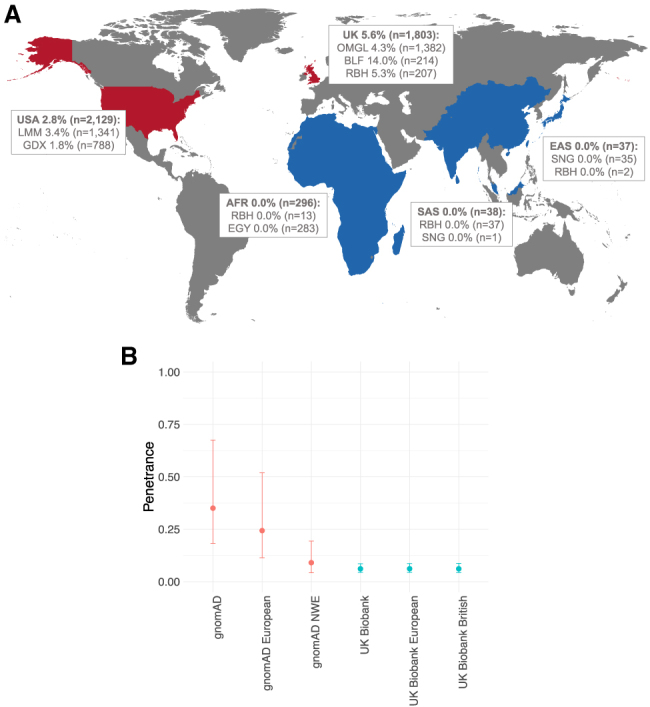
Penetrance estimates are inflated with underestimated population frequency (A) The map of the world emphasizes the large proportion of observations of *MYBPC3* c.1504C>T (p.Arg502Trp) in HCM-affected individuals of Northwestern European (NWE) ancestry. The numbers on the map are the counts of rare-variant-genotype-positive observations (n ≈ cohort participants) from each cohort with the specified ancestry, and the percentages derive the proportion of observations that are due to the *MYBPC3* c.1504C>T (p.Arg502Trp) variant. (B) The graph shows the estimated penetrance and 95% confidence interval for the variant on the basis of subgroups of reference dataset participants included. The penetrance is inflated when estimated with gnomAD because the variant is most common in participants with NWE ancestry (which dominates the UKBB dataset). Population frequency of gnomAD, UK Biobank, and Ensembl population genetics showed that this variant (rs375882485) is only found multiple times in NWE ancestry sub-cohorts. The map excludes Antarctica for figure clarity. A limitation is the low sample sizes for AFR, SAS, and EAS ancestries.^26^

### Clinical impact of specific variants now shown to have low penetrance

We can define the upper bound of the penetrance estimate for some variants. 162 rare variants in HCM-associated genes (63% of variants, observed 745 times [7% case frequency; 17% of observations]) have a penetrance of ≤10%, according to the upper limit [UCI] of the 95% CI for our estimate. These included two variants previously curated as definitively pathogenic and 25 variants curated as likely pathogenic.

One of the pathogenic variants is splice acceptor *MYBPC3* c.26−2A>G (GenBank: NM_000256.3), which has an estimated penetrance of 1.0% (0.4%–2.8%) or 0.9% (0.3%–2.5%) in EUR ancestry, as it was identified four times in EUR affected individuals and 20 times in population participants (90% were EUR). The potential for this variant to have incomplete penetrance has been noted previously through identified asymptomatic individuals with variants (see ClinVar ID 42644). There is *in silico* evidence of an alternate splice site downstream that could result in an in-frame deletion of two amino acids.

The second pathogenic variant identified with a UCI of ≤10% is the missense variant *MYH7* c.3158G>A (p.Arg1053Gln) (GenBank: NM_000257.4), which is a Finnish founder mutation. This variant had an estimate penetrance of 2.2% (0.9%–5.2%), as it was identified seven times in EUR affected individuals and 17 times in the population cohort (16 Finnish from gnomAD, one NWE from UKBB). Estimates of penetrance are sensitive to allele frequency differences across ancestries. Analysis of founder mutations in the population they derive from would provide additional confidence in their penetrance estimates.

For DCM, 17 rare variants (29% of variants) observed 45 times (2% case frequency; 5% of observations) met this criterion. None of the 17 variants were curated as P/LP.

### Penetrance estimate simulations of increased cohort sizes

We anticipate two benefits to estimating the penetrance of rare variants from increasing cohort sizes: (1) there will be more variants that are observed recurrently in affected individuals and populations, permitting AF estimates and hence penetrance estimates, and (2) the precision of our penetrance estimates will increase as AF of rare variants is ascertained with greater precision.

We sought to understand whether it would be more valuable to focus resources on aggregating data from larger numbers of affected individuals (∼100,000 plausible affected individuals with global collaboration efforts), and/or from larger numbers of population participants with near-term publicly available population datasets (∼5,000,000 participants).

Efforts to increase reference population sample size will provide additional confidence in penetrance estimates once case aggregation to 10,000 affected individuals is reached (Figure S6). There is substantial confidence to be gained by increasing the population cohort size: we found that increasing the population dataset from 300,000 participants to 4.5 million participants could provide ∼20% certainty, depending on the penetrance of the variant (Figures S7–S11). The increase in confidence gained from increasing the case cohort sample size from 10,000 affected individuals to 100,000 affected individuals was limited (with the caveat that more variants will be identified).

## Discussion

We show that some subgroups of rare variants in the population are penetrant and for these it may be reasonable to return as SFs. These include ultra-rare variants, predicted PTCs in certain genes where loss of function is a known disease mechanism, and variants with enough evidence to have been classified previously as definitively pathogenic.

There is still uncertainty regarding the penetrance of individual ultra-rare variants, and the implications of returning SFs in healthcare systems have yet to be estimated. While we have previously attempted to assess the burden of long-term surveillance for DCM,^5^ cost-effect analyses are vital to fully understand the risks and benefits of reporting SFs in different healthcare systems. For variant types with low penetrance, it is very uncertain that the benefit of returning SFs will outweigh harms and justify costs.

Here, we provide at-scale estimates of variant-specific penetrance for variants in CM-associated genes that include those likely to be most frequently identified as SFs. Most have low estimated penetrance, where an asymptomatic individual without family history of disease may choose no or less-frequent surveillance depending on the healthcare system and follow-up cost.

Population penetrance estimates derived from unselected individuals (with certain caveats^54^) that are agnostic to personal or family history of disease should provide a better estimate of the probability of manifesting disease when a variant is identified as an SF. Importantly, the penetrance of variants found in individuals with CM and relatives in a clinical setting is increased compared to the penetrance of variants estimated for those identified through SFs (e.g., *MYBPC3* c.1504C>T [p.Arg502Trp] [GenBank: NM_000256.3] with estimated penetrance of 50% in individuals with HCM and 6% here in the population).

While published data are sparse and heterogeneous, overall estimates of penetrance by adulthood in the general population are lower than family-based studies. We used unpublished data to assess the penetrance of asymptomatic individuals with variants referred to hospital for predictive testing after identification of a genotype- and CM-positive relative. For HCM, 17 of 65 individuals with variants (26.2%) were diagnosed with HCM (ten on first clinical evaluation, seven during 2 years of follow up). For DCM, two of 22 individuals with variants (9.1%) were diagnosed with DCM (two on first clinical evaluation, 0 during 2 years of follow up [excluding five with hypokinetic non-dilated cardiomyopathy and four with isolated left ventricular dilatation]). Additionally, a study of individuals with variants identified during family screening who did not fulfill diagnostic criteria for HCM at first evaluation identified HCM or an abnormal ECG in 127 of 285 individuals with variants (44.6%; 82 at baseline, 45 over a median of 8 years follow-up).^25^ First degree relatives in the same household may be at increased risk of disease due to shared environment and other genetic factors.

The ACMG guidelines for reporting “medically actionable” variants in 78 genes come with the caution that evaluating SFs requires an increased amount of supportive evidence of pathogenicity given the low prior likelihood that variants unrelated to the indication are pathogenic.^55^ Here, we show that variants with a definitive pathogenic assertion in ClinVar had the highest penetrance estimates. This may be because penetrant variants are more likely to yield sufficient evidence for confident interpretations, especially family segregation data.

Genetic laboratories communicate their confidence on whether a variant has a role in disease (i.e., pathogenicity) but do not consistently indicate the penetrance. Pathogenicity addresses whether a variant explains the etiology of an individual with disease. In comparison, penetrance addresses the probability of future disease in individuals with variants. The ClinGen consortium Low-Penetrance/Risk Allele Working Group recommends providing penetrance estimates on clinical reports (aggregate gene-level or individual variants) and noting when penetrance is assumed or where current information is limited/unavailable.

Individually rare TTNtvs are collectively common in the general population (∼1 in 250 for variants in exons constitutively expressed in the adult heart; likely due to the size of *TTN* and only moderate constraint [loss-of-function observed/expected upper bound fraction (LOEUF) of 0.35 in gnomAD]), and we show that the penetrance in aggregate of TTNtvs is reduced compared to predicted loss-of-function variants in other CM-associated, haploinsufficient genes. While recent work has increased our understanding of the functional mechanisms of TTNtvs in disease,^56^^,^^57^ future work is required to identify modifiers of TTNtvs to understand this reduced penetrance in the population.

The penetrance of a variant may depend on characteristics of the variant itself and modulating effects of genetic background and environment. This study characterizes individual variants, while ongoing work is dissecting the role of secondary genetic influences. Polygenic scores may identify individuals at particular risk of disease, modifying the estimated penetrance of a single dominant variant.

We present two dimensions to estimates of penetrance: the penetrance in the general population and variant-specific penetrance. As described, the results of this method are concordant with previous population estimates of aggregate penetrance in the UKBB population derived with independent approaches, providing confidence in the methods. In addition, we provide updated estimates for the population prevalence of HCM and DCM and stratify by sex. The addition of future, publicly available, large-scale, global population datasets and biobanks will aid this area of research by allowing for increased confidence in ancestry-specific population allele frequencies and CM prevalence. We provide the summary counts for each variant via an online browser and the function to estimate penetrance in R for transferability and use in other diseases and datasets.

### Limitations

This study has not been undertaken without careful consideration of the limitations. This method cannot quantify the penetrance of pathogenic variants that are absent/singleton in the population, while in aggregate the penetrance of this group of variants is significant.

Comparisons of case and control allele frequency are vulnerable to confounding by population stratification, and we have explored some examples in this manuscript. We do not have genome-wide variation data to directly assess genetic ancestry for the case cohort, so this is based on data reported by the referring clinician. As the EUR participants dominate our case and population datasets, greater representation of diverse ancestral backgrounds is essential for equitable access to genomic medicine. Estimates of the penetrance of variants and the prevalence of cardiomyopathies in more ancestral groups are required. The current data for both comes from UKBB, which has limitations.^54^

In the absence of genome-wide data, we cannot exclude the possibility of unrecognized or cryptic relatedness within the case cohort. As described by Minikel et al.,^26^ when a variant is highly penetrant, cryptically related individuals are likely included in case series and, if a disease is fatal, population cohorts are likely depleted of causal variants.

Case allele frequency in unrelated affected individuals may not be a fair estimate of the case allele frequency in all cases observed in the clinic. Our estimate of case allele frequency, and therefore of penetrance, is influenced by genetic testing referral practice. If clinicians are cautious and only refer selected high confidence affected individuals for testing, case allele frequency and estimated penetrance will be high, whereas if clinicians were to test widely and indiscriminately, then our apparent case allele frequency would be lower, resulting in lower penetrance estimates.^18^

Current diagnostic data assume that the testing center obtained complete coverage of the gene. Limited data were available on age and sex for large portions of the case cohorts. Our DCM-referred cohort was only moderate in size, and thus increases in sample size here through global collaboration would aid our estimates of penetrance for variants in DCM-associated genes. We have estimated penetrance for rare variants that are reported by diagnostic laboratories and have not estimated penetrance for more common variants of smaller effect that may contribute to risk in combination.

Finally, the UKBB volunteer population cohort is healthier than the average individual,^54^ and the gnomAD consortium includes some individuals with severe disease but likely at a frequency equivalent to or lower than the general population.^31^ The proposed penetrance model is an approximation since in reality the three parameters used on the right-hand side of the penetrance equation share some degree of dependence.

### Conclusion

We present an evaluation of the penetrance of individual rare variants in CM-associated genes at scale. These recurrent variants are those that are likely to generate SFs. Variants previously annotated as pathogenic, loss-of-function variants in specific genes susceptible to haploinsufficiency, and those that are the rarest in the population, have high penetrance, similar to observations from family studies. This initial attempt at estimating the penetrance of rare variants has highlighted the requirement for large case and population datasets with known genetic ancestry. We are now able to start putting bounds on the estimate of penetrance for a specific variant identified as a secondary finding: for some, including those expected to be most penetrant, we do not currently have enough data; for others, we can provide asymptomatic individuals with variants with an estimated probability of manifesting disease.

## Data Availability

All case cohort data arising from this analysis is available through DECIPHER (https://www.deciphergenomics.org/). Both gnomAD (https://gnomad.broadinstitute.org/) and UK Biobank (https://www.ukbiobank.ac.uk/) population reference datasets are publicly available. Analysis code is available on GitHub (https://github.com/ImperialCardioGenetics/variantfx/tree/main/PenetrancePaper).

## Supplemental information

Document S1. Figures S1–S14, Tables S3 and S4, supplemental notes, and supplemental methods

Figure S15. An interactive widget for browsing the individual variants in Figure 4 Variant-specific estimates of penetrance for the 316 recurrently observed rare variants in CM-associated genes from group 2. The variants depicted (HCM n = 257 (top), DCM n = 59 (bottom)) were identified multiple times in cases and population reference datasets and penetrance could therefore be estimated. Presented is the estimated penetrance and 95% confidence interval. The x axis denotes the number of times the variant was observed in each case cohort. AC, allele count; B/LB, benign/likely benign; VUS, variant of uncertain significance; LP, likely pathogenic; P, pathogenic

Data S1. Tables S1, S2, and S5–S20

Document S2. Article plus supplemental information
